# Measurement versus prediction in the construction of patient-reported outcome questionnaires: can we have our cake and eat it?

**DOI:** 10.1007/s11136-017-1720-4

**Published:** 2017-11-02

**Authors:** Niels Smits, L. Andries van der Ark, Judith M. Conijn

**Affiliations:** Research Institute of Child Development and Education, University of Amsterdam, Nieuwe Achtergracht 127, 1018 WS Amsterdam, The Netherlands

**Keywords:** Test construction methods, Measurement, Prediction, Predictive validity

## Abstract

**Background:**

Two important goals when using questionnaires are (a) measurement: the questionnaire is constructed to assign numerical values that accurately represent the test taker’s attribute, and (b) prediction: the questionnaire is constructed to give an accurate forecast of an external criterion. Construction methods aimed at measurement prescribe that items should be reliable. In practice, this leads to questionnaires with high inter-item correlations. By contrast, construction methods aimed at prediction typically prescribe that items have a high correlation with the criterion and low inter-item correlations. The latter approach has often been said to produce a paradox concerning the relation between reliability and validity [1–3], because it is often assumed that good measurement is a prerequisite of good prediction.

**Objective:**

To answer four questions: (1) Why are measurement-based methods suboptimal for questionnaires that are used for prediction? (2) How should one construct a questionnaire that is used for prediction? (3) Do questionnaire-construction methods that optimize measurement and prediction lead to the selection of different items in the questionnaire? (4) Is it possible to construct a questionnaire that can be used for both measurement and prediction?

**Illustrative example:**

An empirical data set consisting of scores of 242 respondents on questionnaire items measuring mental health is used to select items by means of two methods: a method that optimizes the predictive value of the scale (i.e., forecast a clinical diagnosis), and a method that optimizes the reliability of the scale. We show that for the two scales different sets of items are selected and that a scale constructed to meet the one goal does not show optimal performance with reference to the other goal.

**Discussion:**

The answers are as follows: (1) Because measurement-based methods tend to maximize inter-item correlations by which predictive validity reduces. (2) Through selecting items that correlate highly with the criterion and lowly with the remaining items. (3) Yes, these methods may lead to different item selections. (4) For a single questionnaire: Yes, but it is problematic because reliability cannot be estimated accurately. For a test battery: Yes, but it is very costly. Implications for the construction of patient-reported outcome questionnaires are discussed.

## Introduction

Both in medical research and clinical practice, Patient-Reported Outcomes (PROs) are increasingly used to obtain information about the physical, mental, and social well-being as experienced by patients. PRO questionnaires may be used for two goals: measurement and prediction. Most often, clinicians and researchers are interested in measurement; that is, the patients’ sum scores accurately represent the patients’ attributes. For example, the KIDSCREEN [4] is a popular questionnaire that is used for assessing and monitoring Health-Related Quality of Life (HRQoL) in children and adolescents. For measurement purposes, the reliability of the sum score is key, because a higher reliability implies more precise measurement. Sometimes, questionnaires are used for predictive purposes; that is, the patients’ sum score is employed to provide a forecast of an external outcome. This outcome may be a future state or behavior that is clinically relevant [5], but may also be the gold standard (i.e., the best measure available [6]) of the concept the questionnaire aims to measure [7]. For example, Foster et al. [8] developed an inventory to assess patients’ need for a functional assessment and used it to predict future utilization of medical services. For prediction purposes, the predictive validity of the sum score is key because a higher predictive validity implies more accurate prediction.

Whether a PRO questionnaire is primarily used for measurement or prediction seldom affects the choice of method to construct the questionnaire (see, e.g., [9–11]). There is an array of construction methods, henceforth called *popular construction methods*, that are used for the construction of all types of questionnaires: Classical Test Theory (CTT, [1–3]), which includes popular methods such as investigating descriptive item statistics, Cronbach’s alpha (e.g., [12]), and the corrected item-total correlations; exploratory and confirmatory factor analysis [13], which include the investigation of the dimensionality of the item scores and the particular item loadings; and item response theory [14] which includes the investigation of item discrimination, item difficulty, and item bias. For example, for the construction of PRO questionnaires, which are primarily used for measurement, the guidelines of the PROMIS initiative [15] prescribe the use of CTT, factor analysis, or item response theory for item selection. The same methods are also used for constructing PRO questionnaires that are used for prediction (e.g., [8]).

From the theoretical work of Lord and Novick [2], we know that the popular construction models are appropriate for constructing questionnaires that are used for measurement, and we know that the popular construction models are suboptimal for constructing questionnaires that are used for prediction.^1^ This seems paradoxical because most of us have learned that good measurement is a prerequisite for good prediction. Some authors proved this assumption to be false empirically. For example, for the construction of the short version of the Screener and Opioid Assessment for Patients with Pain-Revised, Finkelman et al. [18] used construction methods to optimize prediction. They showed that focusing on predictive validity resulted in a substantially lower reliability but equal predictive validity. Similarly, the short version of the Mood and Anxiety Symptoms Questionnaire [19] retained its predictive validity in spite of a drop in reliability. Still, the need for different construction methods for prediction and measurement seems to go unnoticed, leading to the following four questions: (1) Why are the popular construction methods suboptimal for questionnaires that are used for prediction? (2) How should one construct a questionnaire that is used for prediction? (3) Do questionnaire-construction methods that optimize measurement and prediction lead to the selection of different items in the questionnaire? (4) Is it possible to construct a questionnaire that can be used for both measurement and prediction?

In this paper, we first explain why popular construction methods are appropriate for questionnaires that are used for measurement but suboptimal for questionnaires that are used for prediction. Second, we present a method for constructing questionnaires that are used for prediction. By means of an empirical example, we show that the type of construction methods matters. Finally, we discuss the issue whether a questionnaire can be good at both prediction and measurement. In the discussion, we also explain the paradoxical situation that, on the one hand, we intuitively feel that good measurement is a prerequisite of good prediction, whereas on the other hand we apparently need construction methods for both goals that produce different questionnaires.

## Why are the popular construction methods suboptimal for questionnaires that are used for prediction?

This question is answered in three steps. First, we explain the concept of reliability, which is important for precise measurement. Then, we explain the concept of predictive validity, which is important for prediction. Finally, we show the effect of popular construction methods on the estimates of reliability and predictive validity.

### Reliability

In CTT, sum score $X$ consists of a true score $T$ and measurement error *E*:

1 $$\begin{aligned} X =T +E. \end{aligned}$$

$X$, $T,$ and $E$ are conceived in a thought experiment in which for a fixed respondent measurement $X$ is replicated a large number of times (assuming independent trials, identical settings, and no change in the attribute over time), $T$ is the average of this sequence, and random variable $E$ is the error of measurement on an arbitrary measurement occasion [2, pp. 29–30]. In practice, replications under these circumstances are impossible. Therefore, $T$ and $E$ are unobservable and we typically have only one sum score $X$ per respondent. In this context, reliability is defined as the consistency (or reproducibility) of the sum score over replications [20, p. 105]; reliability is high when $E$ is typically small compared to $T.$

Primarily, test theory is concerned with individual differences, and therefore the reliability of a test is determined for a population of respondents rather than a single respondent. Equation (1) is therefore generalized towards a population in which $T$ varies among individuals and reliability is defined as the squared correlation between the sum score and true score. Let $\rho _{X T}$ be the correlation between the sum score $X$ and true score $T,$ and let $\sigma ^2_T$ and $\sigma ^2_X$ be the variance of the true score and sum score, respectively; then the reliability is

2 $$\begin{aligned} \rho ^2_{X T}=\frac{\sigma ^2_T}{\sigma ^2_X}. \end{aligned}$$

Because true scores are unobservable, Eq. (2) is a theoretical definition of reliability [21]. Another step is needed to estimate reliability in practice. CTT introduces an additional measurement $X^\prime,$ which is *parallel* to measurement $X,$ meaning that they have identical true scores $T =T ^\prime$ and variances. It can be shown [2, p. 58] that the squared correlation presented in Eq. (2) is equal to the correlation between these two measurements:

3 $$\begin{aligned} \rho ^2_{X T}=\rho _{X X ^\prime }. \end{aligned}$$

Estimation of reliability therefore reduces to obtaining parallel measurements and calculating correlations among them. The most popular estimator of reliability, Cronbach’s alpha ($\alpha$), consists of a similar approach in situations with more than two parallel measurements; usually item scores $X_i$ ($i=1,2,\ldots , n$). Hence, every item is considered a measurement. Let $\sigma _i^2$ and $\sigma _i$ be the variance and standard deviation of item score *i*, respectively, and let $\rho _{ij}$ be the correlation between item *i* and item *j*. Cronbach’s alpha is defined as

4 $$\begin{aligned} \alpha =\frac{n}{n-1}\times \left( 1-\frac{\sum \nolimits ^n _{i=1}\sigma ^2_i}{\sum \nolimits ^{n}_{i=1}\sum \nolimits ^{n}_{j=1}\sigma _i\sigma _j\rho _{ij}}\right) \end{aligned}$$

(cf. [2, par. 15.3]). Note that in Eq. (4) the unobservable variables have disappeared, and Cronbach’s alpha can be readily computed. If all items are parallel measures, Cronbach’s alpha equals the reliability [21]. If items are not parallel, which is the case in practice, then Cronbach’s alpha is less than the reliability. Equation (4) shows that, all other things being equal, the reliability estimated by Cronbach’s alpha is high when the inter-item correlations are high. In test construction, reliability is therefore optimized by selecting items that show high inter-item correlations. As a result of this approach, the final set of items usually “measure the same thing” [2, p. 95], and the sum score is therefore generally meaningful and interpretable.

### Predictive validity

When constructing a test with a prediction goal, one is interested in the predictive validity of the sum score; that is, the correlation between the sum score and a criterion $Y.$ Let $\rho _{iY}$ be the correlation between the score of item *i* and criterion $Y;$ then the predictive validity equals

5 $$\begin{aligned} \rho _{X Y}=\frac{\sum \nolimits ^{n}_{i=1}\sigma _i\rho _{iY}}{\sqrt{\sum \nolimits ^{n}_{i=1}\sum \nolimits ^{n}_{j=1}\sigma _i\sigma _j\rho _{ij}}} \end{aligned}$$

(cf. [2, Eq. 15.4.4]). The numerator in Eq. (5) shows that, all other things being equal, the predictive validity increases as the item-criterion correlation increases. The denominator in Eq. (5) shows that, all other things being equal, the predictive validity increases as the inter-item correlations decrease. Predictive validity is therefore optimized by selecting items into the questionnaire that correlate highly with the criterion but lowly with the other items (also see, [22, p. 645]). It may be noted that in multiple regression analysis, the same requirements hold: the predictors (a.k.a. independent variables) should be highly correlated with the criterion (a.k.a. dependent variable), and each predictor should have low correlations with other predictors (e.g., [3, chap. 8]). Because this approach results in a final set of items possibly having little relationship among them, the sum score may lack meaningfulness and interpretability [20].

### Effect of popular construction methods on the reliability and predictive validity

Equations (4) and (5) show that measurement and prediction goals have different requirements for inter-item correlations: For measurement, they should be high, and for prediction they should be low. All popular construction methods tend to select items that are highly correlated with the other items in the questionnaire. So all popular construction methods optimize the estimated reliability of the sum score and therefore favor measurement over prediction. For example, if Cronbach’s alpha itself is used as a construction method, then items are selected into the questionnaire that produce the highest value for Cronbach’s alpha. Equation (4) shows that maximizing alpha tends to maximize the inter-item correlations as well and therefore tends to reduce the predictive validity (Eq.  5). From a measurement perspective, maximizing inter-item correlations seems reasonable: If the items that have already been selected to measure the attribute of interest, then an item that correlates highly with the selected items is more likely to measure the attribute than an item that has low correlations with the selected items. For prediction, other construction methods are required.

## How should one construct a questionnaire that can be used for prediction?

Irrespective of the goal of the questionnaire, the construction of the questionnaire, ideally, consists of the following first steps (e.g., [20]). First, the test constructor writes many items for which he or she believes that the item response is indicative for the attribute that the questionnaire should measure. Several conceptual frameworks have been proposed for item writing (e.g., [21, chap. 3]). For example, one can use an explicit theory about the construct [23], or one can use the intuitive knowledge of experts and patients as a basis [24]; what framework is chosen depends on the availability of theories about the construct. Second, in a pilot study, the items are reviewed by a panel of experts and a small sample of respondents, so as to remove the items that are deemed to be of low quality (e.g., when item wording is incomprehensible or offensive). Third, the remaining items, henceforth called the *pretest items*, are administered to a large sample of respondents. The final selection of items, henceforth called the *final items*, is based on the respondents’ item scores, so as to separate the items of high quality from the items of poor quality. Here is when the purpose-specific construction methods come in.

The selection of the final items from the pretest items is a complex task, both for measurement and prediction purposes. First, if the number of pretest items becomes large, the number of potential combinations of final items, $$\sum _{i=1}^n \left( {\begin{array}{c}n\\ i\end{array}}\right),$$ can be huge. For example, for $n =10$ pretest items, there are 1023 possible combinations of one or more final items; for $n =20,$ this number has increased to 1,048,575, and for $n = 40$ to a number that exceeds one trillion ($10^{12}$). For ten pretest items, all subsets of final items can be evaluated, but for more pretest items it becomes unfeasible. Second, the number of automatic subset selection methods, such as forward or backward search algorithms, is large [25]. Third, this huge number of possibilities often exceeds the sample size and may lead to chance capitalization [26].

Therefore, robust and straightforward test construction methods are needed. For measurement purposes, a quick search on Google Scholar shows that many authors use *alpha if item deleted* ($\alpha _{-i}$) (for a discussion see, Raykov [27, 28]): for each item, Cronbach’s alpha is computed with the item removed. Alpha if item deleted can be used in a stepwise backward selection procedure. In the first step, the item with the highest $\alpha _{-i}$ is removed. Next, $\alpha _{-i}$ is computed on the remaining items, and the item with the highest $\alpha _{-i}$ is removed. These steps continue until the desired number of final items has been reached, or earlier, if Cronbach’s alpha has reached the minimal value that is deemed sufficiently high (although various popular heuristic rules of thumb for alpha exist, such as [26], there is no single generally accepted rule; for an overview of suggested rules, see Oosterwijk et al. [29]).

A construction method for selecting final items from a set of pretest items for prediction may follow the same rationale, and the estimated predictive validity can be used in a backward selection procedure:^2^ for each item, the predictive validity with the item removed $\rho _{X_{-i}Y}$ is estimated, and the pretest item with the highest estimated $\rho _{X_{-i}Y}$ is removed. This continues similarly to the backward selection procedure for Cronbach’s alpha. The procedure stops if the desired number of items has been reached, or earlier, if the estimated predictive validity has reached the minimal value that is deemed sufficient for good prediction (there are no widely accepted heuristic rules of thumb for predictive validity; requirements depend on the clinical domain [30], other information available to the tester, and the utility of the outcomes [31]).

Equation (5) shows that for a good predictive validity the final items should correlate highly with the criterion and lowly with the remaining final items in the questionnaire. This means that items probably measure different aspects of the attribute of interest or even more than one attribute.

## Do questionnaire-construction methods that optimize measurement and prediction lead to the selection of different items in the questionnaire?

This question is answered by an empirical example. The data consist of the responses of 242 patients on 10 Likert scale items from the questionnaire Center of Epidemiological Studies-Depression (CES-D, [32]), a self-report inventory consisting in a total of twenty items with a four-point Likert scale which aims to measure depression severity. All respondents also had a criterion score: A binary depression diagnosis on the basis of the Mini International Neuropsychiatric Interview (MINI, [33]), which is often employed as the gold standard. For a more detailed description of the data, the reader is referred to Smits et al. [34]. To keep the illustration general, the CES-D inventory and MINI diagnosis are referred to as ‘questionnaire’ and ‘criterion,’ respectively; the questionnaire items were randomly ordered and are referred to as Item 1, Item 2, et cetera. The full version of the questionnaire had a reliability (estimated using Cronbach’s alpha) of 0.93 and a predictive validity (i.e., the correlation between the sum score and criterion) of 0.40.

The main goal of the illustration is to imitate a test construction situation in which the constructor is faced with a starting set of items, which has previously been administered in a field test or a pilot study, preliminarily to the construction of the final version of the test. The starting set contains more items than can be administered during testing; therefore, a subset of items should be selected. For illustrative purposes, ten items rather than the full set of twenty CES-D items were used. The ten items were selected in such a way that the final set of items showed sufficient variability in the statistics associated with prediction and measurement, which was required for a proper imitation of a pretest situation.

From the pool of ten pretest items, five final items were selected in two conditions. In the first condition, which corresponds to constructing a questionnaire for measurement purposes, the five final items maximized Cronbach’s alpha. In the second condition, which corresponds to constructing a questionnaire for prediction purposes, the five final items maximized the correlation between the sum score and the external criterion. In both conditions, a stepwise backward search procedure, as described in the previous section, was applied for item selection.

Table 1 provides several item statistics for a first inspection of the pool of ten items. Pearson’s inter-item correlations (Table 1, columns 1–9) ranged between .10 and .57, and showed substantial variability. In practice, the pool of items will be larger than 10, and for larger number of items it may be arduous to inspect all inter-item correlations. The corrected item-total correlation, the correlation of an item score with the sum score of the remaining items, may be used instead. The corrected item-total correlations conveniently summarize the inter-item correlations into one statistic per item. The corrected item-total correlations (Table 1, column 10) ranged between .28 and .68, and also showed considerable variability. Items 2, 3, 5, 8, and 10 had the highest corrected item-total correlations. At first glance, these items seem suitable candidate items for a questionnaire that is used for measurement.

**Table 1:** Pearson correlation matrix of ten questionnaire items and a criterion

	Item 1	Item 2	Item 3	Item 4	Item 5	Item 6	Item 7	Item 8	Item 9	Corrected item-total^a^	Criterion
Item 1										0.45	0.22
Item 2	0.32									0.59	0.21
Item 3	0.27	0.50								0.56	0.23
Item 4	0.41	0.31	0.31							0.51	0.28
Item 5	0.27	0.32	0.38	0.34						0.53	0.20
Item 6	0.15	0.42	0.36	0.15	0.30					0.40	0.22
Item 7	0.22	0.38	0.27	0.29	0.31	0.28				0.47	0.26
Item 8	0.36	0.42	0.44	0.42	0.49	0.28	0.44			0.67	0.25
Item 9	0.19	0.19	0.18	0.10	0.20	0.10	0.14	0.33		0.28	0.13
Item 10	0.42	0.46	0.44	0.57	0.41	0.27	0.35	0.55	0.26	0.68	0.35

^a^The sum score of all items except the item in the row; this column provides corrected item-total correlations

The item-criterion correlation (Table 1, last column) ranged between .13 and .35, and showed less variability than the corrected item-total correlations. Items 3, 4, 7, 8, and 10 had the highest item-criterion correlations, but the item-criterion correlations cannot be used directly to assess predictive validity because the inter-item correlations and standard deviations should also be taken into account.

Table 2 (left) shows the five items selected for each condition. For the measurement-based questionnaire, items 2, 3, 5, 8, and 10 were selected; these are the items that showed high item-rest correlations in Table 1. For the prediction-based questionnaire, items 4, 6, 7, 9, and 10 were selected. A salient result is that item 9 was selected for the scale, although item 9 had the lowest corrected item-total correlation and the lowest item-criterion correlation. Apparently, this item explained additional variance of the external criterion. Turning to the comparison of the questionnaires: The two questionnaires consisted of very different final items; only a single item (Item 10) was selected in both questionnaires. The third column of Table 2 shows the estimated reliability of both questionnaires. Both reliabilities are lower than the reliability of the full version of the questionnaire, an outcome in line with the Spearman–Brown prophecy formula (e.g., [2, Eq. 5.1.1]) which states that if a test is split up into parallel subtests, the reliability of each subtest will be lower than that of the full test. Cronbach’s alpha was higher for the measurement-based scale (.80) than for the prediction-based scale (.63); according to some standards (e.g., [35]), the former scale would be a ‘good’ scale, the latter an ‘inadequate’ scale (but neither would be recommended for making high-stakes decisions). The fourth column of Table 2 shows the estimated predictive validity, which was higher for the prediction-based scale (.40) than for the measurement-based scale (.33). It depends on the clinical context how this difference is evaluated; if the test contributes much to utility, even a drop of five percent points in predictive power may mean a large loss [31].

**Table 2:** Results of the measurement-based and prediction-based item subset selection

Scale	Items selected					Coefficient $\alpha$	Predictive validity
Measurement-based	Item 2	Item 3	Item 5	Item 8	Item 10	0.80	0.33
Prediction-based	Item 4	Item 6	Item 7	Item 9	Item 10	0.63	0.40

To evaluate the size of differences in outcomes between the two scales, it may be informative to relate them to the outcomes of the full version of the questionnaire: compared to selecting items for a measurement-based scale, selecting items for a prediction-based scale yields a larger reduction of Cronbach’s alpha: $(1 - .63/.93)\times 100 = 32\%$ versus $(1 - .80/.93)\times 100 = 14\%,$ but a smaller reduction of predictive validity $(1 - .40 /.40)\times 100 = 0\%$ versus $(1 - .33/.40)\times 100 = 18\%.$

This empirical example illustrates that construction methods for measurement-based questionnaires and prediction-based questionnaires may result in different sets of final items, and that a trade-off between measurement and prediction properties of a scale exists. It also illustrates that for optimizing predictive validity a high value of Cronbach’s alpha is no prerequisite.

## Is it possible to construct a questionnaire that can be used for both measurement and prediction?

What would be the merit of a questionnaire with both high measurement and prediction qualities? It would mean that a questionnaire is not only useful for forecasting purposes, but also that its sum scores are meaningful and interpretable, which has at least three advantages. First, such a questionnaire more easily gains acceptance among test users because it can be used for all purposes. Second, predictions based on such tests are more easily communicated to test takers. Third, it allows for studying the mechanisms underlying the relationship between test and criterion, which in turn may allow for refinement of the test.

There are two answers to the question as to whether it is possible to construct a questionnaire that can be used for both measurement and prediction. Both answers indicate that it is possible to construct a questionnaire that can be used for both measurement and prediction, and both answers stress that there is no such thing as a free lunch. The answers require the introduction and the explanation of a paradox.

### The paradox

On the one hand, Eqs. (4) and (5) show that measurement goals require high inter-item correlations, whereas prediction goals require low inter-item correlations. This suggests that questionnaires cannot be used for both measurement and prediction. On the other hand, it can be proven mathematically [2, p. 72] that the predictive validity of the sum score can never be higher than the square root of the sum-score reliability; that is,

6 $$\begin{aligned} \rho _{X Y}\le \rho _{X T}=\sqrt{\rho _{X X ^\prime }}. \end{aligned}$$

Only if criterion $Y$ equals the true score of $X,$ the predictive validity equals the square root of the reliability. In practice, the predictive validity can be expected to be much lower than the square root of the reliability. Equation (6) suggests that a high reliability is a prerequisite of predictive validity. So, we have a paradox: According to Eqs. (4) and (5), predictive validity and reliability do not go well together, and according to Equation 6 they must go together. Several authors have noted this paradox (e.g., [1], pp. 380–381; [2], pp. 332–333, [3], p. 243).

The paradox can be explained by the fact that Cronbach’s alpha (Eq. 4) is an estimate of the reliability and not the reliability itself. Cronbach’s alpha is a lower bound: For all practical applications, Cronbach’s alpha is smaller than the reliability [2].^3^ The crucial part is that the difference between the true reliability and the reliability estimated by Cronbach’s alpha becomes larger as the inter-item correlations decrease. As a result, for questionnaires with low inter-item correlations, Cronbach’s alpha is a poor estimate of the reliability. The Appendix shows that if the reliability equals .8, Cronbach’s alpha can be as low as zero. Hence, the paradox exists because one of the premises uses estimated reliability (Eq. 4), whereas the other premise uses true reliability (Eq. 6).

### The two answers

The first answer pertains to a single questionnaire. The answer is yes, in principle a single test can have a high predictive validity and a high reliability, but in practice it may be problematic. For good predictive validity, a construction procedure may be used that optimizes the predictive validity. These construction procedures tend to select items with low inter-item correlations (Eq. 5), and low inter-item correlations will generally produce low values of the reliability estimated by Cronbach’s alpha (Eq. 4). However, the explanation of the paradox indicated that for questionnaires with low inter-item correlations, Cronbach’s alpha is a poor estimate of the reliability. As a result, the questionnaire may or may not have an adequate reliability.

The major problem is that the reliability cannot be estimated accurately using Cronbach’s alpha or other internal consistency methods, such as Guttman’s lambda coefficients [37]. Alternatives for reliability estimates include the test-retest correlation (e.g., [7]) and reliability estimates that work well for multidimensional data. The test-retest correlation has the advantage that it does not depend on inter-item correlations, but it has the disadvantage that the conditions in the test and retest are assumed to be equivalent [22, 38] (e.g., for the retest, it is assumed that patients do not remember what they answered in the first administration). A violation of the assumption renders the test-retest correlation useless as a reliability estimate: It may overestimate the true reliability (e.g., because patients remembered the answers) or it may underestimate the true reliability (e.g., because the physical conditions in the two administrations were not identical). Reliability estimates that can be used for multidimensional data may be more accurate than Cronbach’s alpha. Examples include reliability estimates based on multiple-factor models [39] or latent-class models [40].

The second answer is also yes, but is it rather costly and time consuming. It requires a test battery rather than a single questionnaire: A test battery is a collection of tests and/or questionnaires which typically measure different variables but which have a common objective [41]. On the one hand, each test and questionnaire is constructed to be a reliable measurement of the attribute it intends to measure. On the other hand, the scores on tests and questionnaires have low correlations, making them suitable for prediction. For example, Perrine and colleagues [42] used the Quality of Life in Epilepsy-89 inventory in combination with intelligence tests and mood questionnaires to study HRQoL in epilepsy. Test batteries have the great advantage that each predictor is carefully constructed. Given that these predictors are selected based on a clear theoretical framework, one does not only predict well, but it is more likely that one understands the prediction as well. (Evidently, the predictive power and interpretability of the battery depend on what is being measured and predicted, and the quality of test items.) Test batteries, however, have the great disadvantage that they are expensive and time consuming with respect to both construction and administration.

## Discussion

We answered four questions:

1. Why are the popular construction methods suboptimal for questionnaires that are used for prediction? Popular construction methods optimize the estimated reliability of the sum score by selecting items that have high inter-item correlations, and we showed that for predictive validity inter-item correlations should be low. In addition, this result indicates that, instead of a property of a questionnaire that can be evaluated after test construction (see, e.g., [9–11]), predictive validity is a property that can be optimized in the construction phase itself; if prediction is the goal, appropriate construction methods should be used.
  Although under item response theory models sum-score reliability may be obtained [43], the concept of internal consistency is replaced by that of item and test information [14]. Information quantifies the measurement precision a test or an item provides as a function of the latent trait (higher information implies more reliability). Therefore, it may seem as if employing this theory secures from the measurement–prediction trade-off. However, this is incorrect because item information is known to be higher for items with high discrimination parameter values [44], and that in practice discrimination parameters are highly correlated with corrected item-total correlations [45]. As a result, item selection optimizing test information is expected to result in item sets similar to those resulting from item selection based on optimizing internal consistency.
2. How should one construct a questionnaire that is used for prediction? This is a question for which we do not have a definite answer. We discussed a stepwise backward selection algorithm for item selection that optimizes the estimated predictive validity: the sum score-criterion correlation. However, this study is no plea for pushing the optimal approach for prediction to the extreme. Evidently, a questionnaire consisting of items that correlate highly with an external criterion, but have little relationship with each other, may lack meaningfulness and interpretability [20, 46]. In contrast, the message is that homogeneous items usually explain the same part of the criterion’s variance, and that by selecting items that have less in common, a larger part of the criterion’s variance may be explained. Moreover, there are limits to this approach, because it follows from Eq. (5) that it is mathematically impossible to find a large set of items with zero correlations among them [2, p.333]. Similarly, in multiple regression analysis, the number of meaningful predictors is usually not more than four or five [2, p. 274].
  In the non-clinical literature on test construction, it has been advised not to construct prediction-based scales because a single well-defined criterion in a specific setting is hardly, if ever, available (e.g., [20, p.323]). Also, for general HRQoL measures, such as the Short Form Health Survey (SF-36) [47], typically no single criterion is available. However, for clinical measures that assess disease-specific patient-reported outcomes well-defined criterion measures are much more common. For example, questionnaires have been constructed to serve as a first assessment in two-stage testing (e.g., [31, 48]). The second test consists of an extensive examination of the individual, often referred to as the gold standard, which results in a classification like ‘healthy’ or ‘diseased.’ The first test, often referred to as ‘screener,’ is a cheap indicator of illness, and only when it gives a positive outcome, the more expensive examination is performed. Examples of such screeners are the Patient Health Questionnaire-Depression (PHQ-9, [49]), Generalized Anxiety Disorder Assessment (GAD-7, [50]), and Pain Numeric Rating Scale (NRS, [51]). Note that in this situation it would be inappropriate to evaluate the questionnaire using Cronbach’s alpha. In contrast, the quality of a screener should be primarily evaluated on the basis of its diagnostic accuracy (including sensitivity, specificity, and predictive values) in the target population [7], and its reliability should be assessed using a method that does not severely underestimate reliability in case of low inter-item correlations. We have argued that these tests are less suited for measurement (also, see [52]).
3. Do questionnaire-construction methods that optimize measurement and prediction lead to the selection of different items in the questionnaire? We showed they do. As a result, there seems to be a trade-off between measurement goals and prediction goals. This trade-off bears a close resemblance to the relationship between homogeneity and breadth of attribute [53–55]. The homogeneity of a questionnaire may be maximized by selecting items that are similar in content, but this usually means a loss of generality of the attribute, that is, a decrease in content validity [56]. Selecting homogeneous items may have a negative effect on both predictive validity and construct validity. Whereas predictive validity is concerned with the relationship with a single criterion, construct validity is concerned with the relationship with a diversity of criteria. Therefore, too much emphasis on homogeneity may be detrimental to a multitude of relationships. For example, Devine and colleagues [57] constructed an item bank for measuring depression using item response theory. Items associated with physical aspects, such as loss of appetite, did not fit the measurement model and were excluded from the item bank. Hence, the homogeneity of the items increased but the breadth of the attribute decreased. Devine et al. compared the sensitivity to clinical change between the questionnaire that used items from the item bank and several legacy instruments that were based on expert knowledge. They found that the legacy instruments were more sensitive to clinical change. Apparently, the increase in homogeneity also affected the predictive validity. A related issue is imbalance of item content: If some subdomains are over-represented in the pretest items, pursuing homogeneity may lead to a final item set that misses important aspects of the attribute. For a discussion, we refer to [34, 58].
4. Is it possible to construct a questionnaire that can be used for both measurement and prediction? It is possible to have the cake and eat it, but at a price. A single test, constructed for prediction, may possibly have a sufficient sum-score reliability. Unfortunately, we would not know because the estimates are too far off, and for the same token, the sum-score reliability may be poor. Measuring and predicting at the same time requires questionnaire batteries (also see [53]). Test batteries are far from the efficiency that is customary in HRQoL. As a final note, we believe it is important that test constructors, editors, and reviewers of scientific journals know that it is hard for a questionnaire to excel on all properties. Moreover, it is important to provide as much information as possible on as many test qualities as possible, but the questionnaire should be mostly evaluated on properties which are associated with the goal for which it was constructed.

## Appendix

Let $\sigma ^2_{T_i}$ be the variance of the true score of item *i*. Because the true scores are unobservable, $\sigma ^2_{T_i}$ is also unobservable. Reliability (Eq. 3) can be written as

A.1 $$\begin{aligned} \rho _{X X ^\prime }=\frac{\sum \sum _{ i \ne j} \sigma _i \sigma _j \rho _{ij} +\sum _i\sigma ^2_{T_i}}{\sum _{i}\sum _{j} \sigma _i \sigma _j \rho _{ij}}, \end{aligned}$$

(cf. [2, Eqs. 4.3.7 and 4.3.8]). Let $\bar{\sigma } =\frac{\sum \sum _{ i \ne j} \sigma _i \sigma _j \rho _{ij}}{n (n-1)}$ be the mean inter-item covariance. Cronbach’s alpha can be rewritten as

A.2 $$\begin{aligned} \alpha =\frac{\sum \sum _{ i \ne j} \sigma _i \sigma _j \rho _{ij} +\sum _i\bar{\sigma }}{\sum _{i}\sum _{j} \sigma _i \sigma _j \rho _{ij}}. \end{aligned}$$

Eqs. (A.1) and (A.2) show that the unobservable part of the reliability, $\sigma ^2_{T_i},$ is replaced by the mean inter-item covariance, $\bar{\sigma },$ to obtain $\alpha.$ Other than that, Eqs. (A.1) and (A.2) are the same. Table 3 (rows 1–3) shows, for two three-item questionnaires, the population values of the item variances, the inter-item correlations, and the item true score variances. Using Eqs. (A.1) and (A.2), the reliability and Cronbach’s alpha are computed (Table 3, rows 4–9). For questionnaire 1, the inter-item correlations are high, whereas for questionnaire 2 the items are uncorrelated. For both questionnaires, the sum-score reliability equals $\rho _{X X ^\prime }=.8.$ For questionnaire 1, the reliability is estimated with $\alpha =.75.$ Although alpha is a lower bound, the difference is relatively small. For questionnaire 2, the reliability is estimated with $\alpha =0.$ Hence $\alpha$ greatly underestimates the sum-score reliability.

**Table 3:** Two questionnaires that have the same sum-score reliability but different values for Cronbach’s alpha

	Questionnaire 1	Questionnaire 2
Item variance (for all items)	1	1
Inter-item correlations	$$\begin{pmatrix}1 &{} .5 &{} .5\\ .5&{} 1 &{} .5 \\ .5 &{} .5 &{} 1\end{pmatrix}$$	$$\begin{pmatrix}1 &{} 0 &{} 0\\ 0&{} 1 &{} 0 \\ 0 &{} 0 &{} 1\end{pmatrix}$$
Item true score variances	.6	.8
$\sum \sum _{ i \ne j} \sigma _i \sigma _j \rho _{ij}$	3	0
$\sum _{i}\sum _{j} \sigma _i \sigma _j \rho _{ij}$	6	3
$\sum _i\sigma ^2_{T_i}$	1.8	2.4
$\sum _i\bar{\sigma }$	1.5	0
$\rho _{X X ^\prime }$	$\frac{3 + 1.8}{6}=.8$	$\frac{0 + 2.4}{3}=.8$
$\alpha$	$\frac{3 + 1.5}{6}=.75$	$\frac{0 + 0}{3}=0$

## Footnotes

1 Also, see Guttman’s theoretical work on scale construction for prediction [16, 17].

2 Finkelman et al. [18] used a similar method for item selection: absolute shrinkage and selection operator (lasso) regression. For a discussion of lasso, see Hastie et al. [25, chap. 3].

3 Only if the item scores are essentially tau-equivalent [36], Cronbach’s alpha equals the reliability. Essential tau-equivalence requires that for all patients and all items, the difference between two item true scores is constant across patients. Although the assumption of essentially tau-equivalence is slightly less restrictive than the assumption of parallel items discussed earlier, it will never hold in practice.
